# The Role of Social Media as a Resource for Mental Health Care

**DOI:** 10.3390/ejihpe13060078

**Published:** 2023-06-10

**Authors:** Ivan Herrera-Peco, Invención Fernández-Quijano, Carlos Ruiz-Núñez

**Affiliations:** 1 Faculty of Health Sciences, Alfonso X el Sabio University, Avda. Universidad, 1, Villanueva de la Cañada, 28691 Madrid, Spain; 2 Faculty of Psychology, Universidad Alfonso X el Sabio, Avenida Universidad, 1, Villanueva de la Cañada, 28691 Madrid, Spain; invenfer@uax.es; 3 Program in Biomedicine, Translational Research and New Health Technologies, School of Medicine, University of Malaga, Blvr. Louis Pasteur, 29010 Málaga, Spain; carlos.ruiz@uma.es

One of the major lessons learned from the COVID-19 pandemic was the importance of caring for the mental health of populations [1], which encompasses the emotional, psychological, and social states of individuals. Therefore, mental health is essential for meaning people’s well-being, and when it is compromised, it affects all aspects of a person’s life [2]. 

Stress or anxiety caused by fast-paced and demanding work environments, as well as high social expectations, may have a devastating effect on people’s psychology. Mental health care for populations and individuals has become critical and increasingly relevant due to the unique challenges we face in the digitization and globalization of information. In this context, it is important to highlight that social media have had a dramatic impact on society, transforming the way we communicate, interact, form groups, and access information.

Social media have emerged as a new environment in which a considerable part of the population invests a great deal of time. We have begun to see social media’s impact on mental health and the way it should be properly used. Social media can be deemed as elements, with both negative and positive effects on people’s mental health [3]. They can be used as a valuable tool to support and promote mental health, but they also carry risks. In this article, we examine these two facets and explore how mental health care and social media use are interrelated.

Some negative effects associated with mental health and the use of social media may include: (i) encouraging constant comparison with others, which is associated with a decrease in self-esteem, (ii) facilitating the harassment of those who think differently and even ridiculing people with mental illness, (iii) the excessive use of social media can lead to dependency and social isolation, affecting the emotional well-being of individuals, and (iv) consuming fake news or biased information can lead to health problems for individuals who accept advice from people who are not qualified to treat their problems or by not seeking help from healthcare professionals out of fear or mistrust [4].

An example of the a harmful effect of social media on mental health is suicide, where social media exposure, parental pressure, or social contagion have been associated with risk factors among young people [5,6]. Hate speech or the misunderstanding of people suffering from mental illness may also occur, such as those diagnosed with schizophrenia. [7] Public derision and a negative portrayal of some aspects of illnesses can lead to worsening the condition. 

On the other hand, social media can be seen as an opportunity to promote mental health care for populations. The large influence they have on the dissemination of health information is highly valuable, both because of the amount of time users spend on them and because of their nature, as sources of accessible and understandable knowledge. Among social media, those of an audiovisual nature, such as Instagram, TikTok or YouTube, are increasingly valuable, since written health information may be phrased in a poorly understandable way or even with minimal scientific support [8].

The potentially positive effects of social media on mental health care include: (i) them being a source of information, providing access to resources, advice and specialized professionals, (ii) people’s participation in online groups or communities, which can provide a sense of belonging, emotional support and the opportunity to share experiences with people facing similar challenges, (iii) them playing a vital role in disseminating information about mental health, raising awareness and reducing the stigma associated with these conditions, and (iv) some applications and platforms have developed digital intervention programs, offering online therapy, self-management tools and emotional support programs. All of these digital resources can reach people who would not otherwise have access to mental health services and can play an important role in the prevention, detection and even treatment of mental disorders.

Thus, despite the risks associated with inappropriate use of social media, there is also evidence that they can play a beneficial role in mental health care. For example, the #chatsafe initiative provides a safe medium for young people aged 10–24 years to communicate online about suicide. It has been found to reduce the risk of distress and future suicidal behavior among young people who attempted suicide or were in contact with people who did [9]. Another interesting example of the potential of social media is the use of short videos focused on minimizing the stigma that patients diagnosed with mental illnesses, such as schizophrenia [10].

Undoubtedly, it is essential to develop research focused on new forms of communication in today’s society and how people form social contact networks from which we obtain information that we can consider to be trustworthy and rely on.

These social media and the information shared within them are essential to mental health care and to the well-being of populations. However, both the potential dangers and benefits of using social media in this context need to be addressed in order to enhance their use for mental health support and promotion. To achieve this, the healthy and critical use of social media must be encouraged, educating users about the risks involved and promoting effective digital interventions that provide adequate resources and support. In doing so, we can maximize their potential as allies in mental health care and contribute to a healthier, more educated, tolerant and emotionally balanced society.

## Data Availability

Not applicable.
